# The MyGuide Web-Based Self-Management Tool for Concussion Rehabilitation: Mixed Methods Cross-Sectional Study

**DOI:** 10.2196/59181

**Published:** 2025-01-07

**Authors:** Alyssa Turcott, Ruthine Kang, Christopher Yao, Colleen O'Melinn, Patricia Mahoney, Susan Barlow, Julia Schmidt

**Affiliations:** 1 Department of Occupational Science and Occupational Therapy Faculty of Science University of British Columbia Vancouver, BC Canada; 2 Rehabilitation Research Program, Centre for Aging SMART Vancouver Coastal Health Vancouver, BC Canada; 3 Canadian Association of Occupational Therapists Ottawa, ON Canada; 4 Vancouver Coastal Health Vancouver, BC Canada; 5 Department of Occupational Science and Occupational Therapy Faculty of Medicine University of British Columbia Vancouver, BC Canada

**Keywords:** concussion, self-management, health information technology, perceptions, concussion recovery, concussion management, concussion rehabilitation, rehabilitation, self-management tool, perception, digital health, e-health, mobile app, mhealth, web-based tool

## Abstract

**Background:**

Web-based concussion self-management education programs for adolescents can improve functional outcomes, reduce concussion symptoms, and increase self-efficacy. However, there are a limited number of studies examining the perceptions and acceptance of these programs and the use of these tools in the adult concussion population.

**Objective:**

This study aimed to investigate the perceptions and acceptance of clinicians and adults with concussions using MyGuide Concussion (Vancouver Coastal Health), a web-based concussion self-management tool.

**Methods:**

Using a mixed methods sequential explanatory design, a convenience sample of 8 adults with concussions and 8 clinicians who used MyGuide Concussion over a 2-year period were interviewed, and their responses were analyzed.

**Results:**

Participants reported two key benefits of using the web-based self-management tool: (1) the tool’s emphasis on the interconnectedness of physical and psychological symptoms, and (2) the ability to provide reassurance that symptom being experienced were a normal part of the concussion experience. Clinicians described the tool as being useful as a supplementary source of information for clients in addition to clinical sessions and believed the content was useful for increasing clients’ independence in managing their own recovery.

**Conclusions:**

Overall, the evaluation of the MyGuide tool is an acceptable and well-perceived tool for adults with concussions who require a basic understanding of concussion recovery, particularly in the early stages of recovery. Future research may include optimizing MyGuide by targeting promotional strategies and addressing other barriers to use.

## Introduction

Lifetime concussion prevalence in adults has been estimated to be as high as 29% [1], with an annual incidence rate of approximately 1.2% in recent years being the highest it has ever been [2]. Concussion symptoms vary between people and across an individual’s recovery process [3]. Symptoms can be experienced as physical (eg, nausea, headaches, and decreased balance), psychological (eg, worsened anxiety, depression), or cognitive (eg, changes in concentration and memory) [4-6]. While most people recover within 2 weeks without intervention, some people experience persistent symptoms [7].

Self-management is a type of educational intervention that provides an individual with strategies and lifestyle behavior changes to manage the injury and improve overall recovery [8]. Concussion self-management (eg, information about concussion symptoms, prognosis, self-management behaviors, and resuming preinjury roles) can improve a person’s understanding of their injury and related symptoms such as sleep and anxiety, reduce postconcussion symptoms [9-11] and facilitate recovery and return to preinjury activities and roles [12-18]. Furthermore, the timely provision of concussion education in the early stages of recovery has been found to lower the risk of developing prolonged postconcussion symptoms, especially for those with more severe traumatic brain injury (TBI), and when implemented within 3 months of injury [13,19]. Though there is no consensus on the optimum time for the introduction of concussion education, studies reporting improvements mainly focused on participants who were between 1 week to 6 months post injury [13,17].

-Technology-based education programs for individuals experiencing concussion symptoms show promise for improving recovery outcomes and self-management self-efficacy, given their accessibility and convenience, particularly for those living in rural and remote areas or areas that do not offer specialized health services [20]. For clinicians, the web-based delivery of health services and education can enhance clinician-client engagement and improve client-driven active participation in their own health [21]. For adults, a self-paced, web-based educational intervention for civilian, military service members, and veterans with postconcussion symptoms was found to increase self-management self-efficacy and improve concussion symptoms for individuals that had longer periods since injury (ie, 1 year) [22]. Overall, the evidence, though limited in amount, supports technology-based concussion education programs as a far-reaching and accessible approach to enable behavioral change, improve recovery outcomes, and help individuals return to their occupations before their injury.

**Figure 1:**
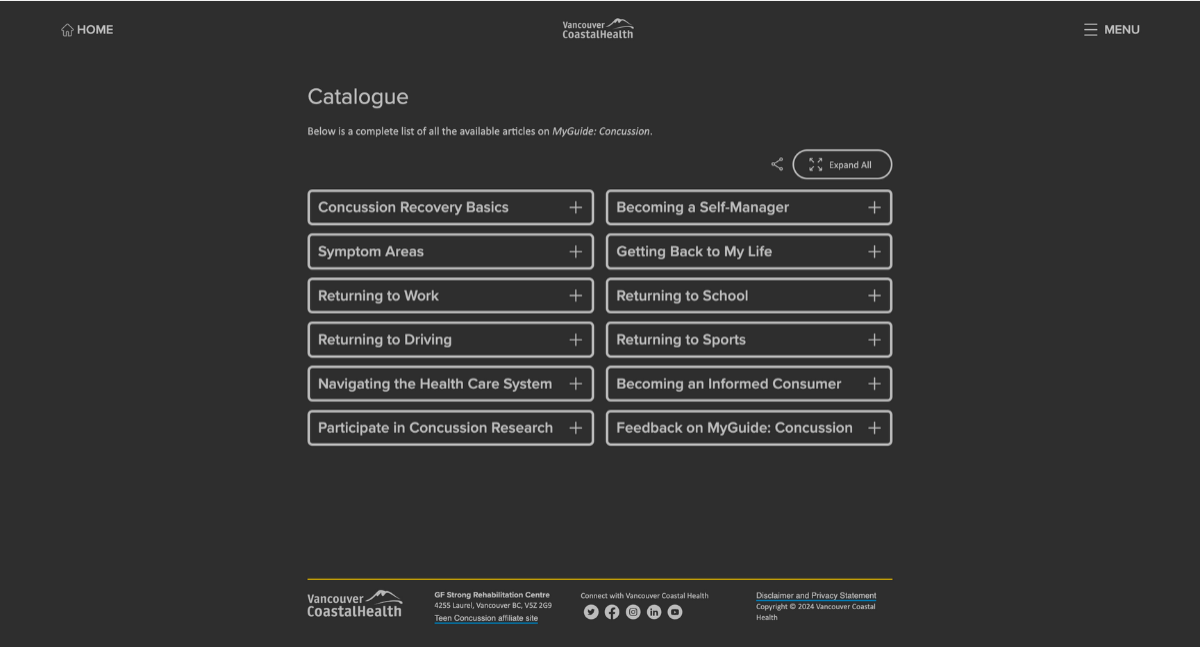
Modules from the MyGuide Concussion tool as they appear in the Catalogue view.

The MyGuide Concussion tool (Vancouver Coastal Health) aims to provide current best practice knowledge to help individuals with a concussion manage their symptoms and empower them with the skills and confidence to take action in their recovery journey. While there are a number of websites that provide evidence-based concussion education, the MyGuide Concussion tool was designed to fill a gap by providing customized material and actionable ways to implement evidence-based self-management strategies into daily life. It will thus be referred to as a self-management tool. The tool was developed in 2019 by experienced clinicians working in the field of concussion, content experts, and through consultation with community stakeholders. The clinical material provided in the MyGuide Concussion tool was based on the Guideline for Concussion/Mild Traumatic Brain Injury & Persistent Symptoms developed by the Ontario Neurotrauma Foundation [23], and information is updated every 6 months. The MyGuide Concussion tool creates a personalized guide for each user, specifically targeting adults (ages 18 years and older) at any stage of recovery. Content is based on modules that include information, tools, and worksheets on relevant topics for people with concussions, as shown in Figure 1. The tool provides reminders to facilitate positive health behaviors and promote self-management while engaging with the tool (eg, taking regular breaks). Though intended to be used on a desktop computer, the MyGuide Concussion tool can be used on any device that can access the internet site.

Much of the literature on technology-based interventions involves randomized controlled trials examining postconcussion symptom improvement after interventions; however, in addition to quantitative measures, understanding the perceptions and acceptance of self-management tools is essential to identify barriers and facilitators to adherence and effectiveness of these tools. In a study on digital health interventions for concussion in adults, the perceptions of the intervention were positive, with high overall usability [24]. Another study implemented a web-based concussion education program and indicated that the concussion education program was easy to use and helpful for participants’ recovery [25]. Overall, the perceptions and acceptance of web-based concussion education programs for adults is a relatively underexplored area, and it is important to continue to explore the design considerations that may be unique to this particular population. Importantly, the perceptions of the MyGuide Concussion tool have not yet been investigated.

The main purpose of this study was to evaluate the perceptions and acceptance of the MyGuide web-based customizable concussion self-management tool in the adult population. Perceptions will be determined based on usability, accessibility, and suitability of the site, and acceptance will be evaluated based on adherence to and impact and relevance of the site.

## Methods

### Study Design

This study used a mixed methods sequential explanatory design [26]. Integration of quantitative and qualitative data occurred at the level of sampling and analysis [27]. For sampling, we used a connecting approach in that participants who completed the quantitative surveys were invited to participate in the qualitative interviews. For the analysis, we used a merging approach, in that we compared responses from the surveys to comments from the interviews to identify any differences and gain a deeper understanding of the perceptions and acceptance of the MyGuide Concussion tool. Qualitative interview questions were developed based on responses provided in the quantitative surveys. The Design and Evaluation of Digital Health Interventions (DEDHI) framework was used to develop survey and interview questions in order to understand the perceptions and acceptance of using MyGuide. The DEDHI framework is a consolidation of various health promotion evaluation frameworks and life cycle models specific to digital health programs and can be used in all aspects of the technological life cycle, from early conceptual models to large-scale implementation in the health care sector [28].

### Quantitative Recruitment

Recruitment occurred from December 11, 2020, to May 31, 2021, using convenience sampling. Recruitment for the survey was done through a pop-up window that appeared on the MyGuide Concussion tool. Individuals with concussion were included in the survey if they were 19 years or older, experienced a concussion within the last 2 years, and used the MyGuide tool. Individuals with any neurological health condition (eg, stroke, multiple sclerosis, Parkinson, and spinal cord injury) that may require services and resources beyond the scope of the tool were excluded from the study.

### Quantitative Data Collection

A total of 8 participants with concussions completed a 10- to 15-minute survey conducted online or by telephone from January 24, 2021, to May 30, 2021. The survey included questions about general demographic information and an outcome measure of technology usability (Health Information Technology Usability Evaluation Scale (Health-ITUES)). This measure consists of 20 items with 4 subscales of impact, perceived usefulness, perceived ease of use, and user control, which have been used to assess the usability of web-based programs for adults living with a chronic health condition [29]. The measure has demonstrated high internal consistency reliability (Cronbach α=0.85-0.92).

### Quantitative Data Analysis

The data are reported using the Good Reporting of a Mixed-Methods Study [30]. Quantitative data were analyzed using descriptive analysis to describe the demographics and Health-ITUES questionnaire.

### Qualitative Recruitment

Recruitment occurred from February 7, 2021, to September 9, 2021. Participants with concussions were invited to participate in the qualitative interviews once they had reached the end of the quantitative survey. Recruitment for clinicians occurred through the use of the researchers’ professional network. Clinicians were included if they were currently working in the concussion rehabilitation field for at least 2 years, had therapeutic roles (eg, occupational therapists, physiotherapists, doctors, and neurologists), were practicing in British Columbia, and were familiar with or used the content in the MyGuide tool.

### Qualitative Data Collection

Participants (both clinicians and people with concussions) engaged in online semistructured interviews through Zoom [Zoom Video Communications] from February 7, 2021, to September 9, 2021. The interview guides for participants with concussions are provided in Multimedia Appendix 1. The interview guides for clinician participants are provided in Multimedia Appendix 2. Interview questions for participants with concussions were adapted from a previous study that examined the user experiences of a self-management tool for individuals with spinal cord injury and their caregivers [31]. Qualitative analysis occurred alongside data collection to ensure data saturation was obtained.

### Qualitative Data Analysis

The qualitative data were analyzed using conventional content analysis. This involves an inductive process of reviewing the data as a whole, then deriving codes from the individual responses themselves and categorizing these to identify connections and relatedness [32]. Conventional data analysis allows researchers to avoid imposing preconceived ideas or categories on participants and, instead, to focus on getting information directly from participants [32].

The qualitative data followed a 2-step analysis process**.** First, qualitative interview data were recorded and transcribed verbatim using a transcription program. Once transcribed, 2 researchers reviewed the transcripts and audio recordings for accuracy and removed any potential identifiers to protect participant identity. Each performed conventional qualitative content analysis separately and then compared to ensure accuracy and resolve any interpretive discrepancies. Both researchers performing the analysis were female undergraduate students, one with a background in behavioral neuroscience and the other in biology and psychology.

The research team used two main trustworthiness strategies. First, the authors were mindful of their positioning throughout data collection and analysis. A diverse range of perspectives was included to establish consensus in data analysis, including the remaining authors following the first round of coding. Complementary perspectives on the data were thus acknowledged, which allowed the authors to reflect on their biases [33]. Second, rich verbatim quotations from the interviews were included in the results to fully capture the voices of participants and increase the credibility of the findings [33,34].

### Ethical Considerations

Ethics approval was completed by the University of British Columbia’s Behavioural Research Ethics Board (H20-02982).

## Results

### Quantitative Results

A total of 8 participants with concussions completed the surveys. Participants with concussion included 4 female and 4 male participants with an average age of 33.8 years (SD 12.3; Table 1). Health-ITUES scores are included in Table 2.

**Table 1:** Demographics of participants with concussions who participated in the interview.

Characteristics		Study participants (N=8)
**Average age in years**		
	Mean (SD)	33.8 (12.3)
	Median (range)	36.5 (21-54)
**Gender, n (%)**		
	Female	4 (50)
	Male	4 (50)
**Race^a^** **, n (%)**		
	White	4 (57)
	Black	1 (13)
	South Asian	1 (13)
	Middle Eastern	1 (13)
**Highest education, n (%)**		
	High school	4 (50)
	College or Bachelor’s degree or higher	4 (50)
**Residence** **, n (%)**		
	BC Greater Vancouver	5 (63)
	Outside of BC Greater Vancouver	3 (37)
**Time since concussion (months), n (%)**		
	<1	3 (37)
	1-3	2 (24)
	3-12	3 (38)
**Mechanism of injury, n (%)**		
	Fall	4 (50)
	Sports injury	2 (25)
	Motor vehicle accident	1 (13)
	Blow to the head	1 (13)
Currently experiencing symptoms, n (%)		8 (100)
Have had previous concussions, n (%)		4 (50)
Full recovery from previous concussions^b^, n (%)		4 (100)
Decreased screen tolerance, n (%)		6 (75)
Receiving Occupational Therapy services, n (%)		1 (13)

^a^One participant preferred not to answer.

^b^Of the 4 participants who had a previous concussions.

**Table 2:** Health-ITUES (Health-Information Technology Usability Evaluation Scale) scores of participants with concussion.

Scale	Mean (SD)
Impact	4.41 (0.46)
Perceived usefulness	4.10 (0.57)
Perceived ease of use	4.42 (0.46)
User control	3.93 (0.45)

### Perceptions: Usability

The Health-ITUES subscales Perceived Ease of Use and User Control were used as indicators of ease of navigation of the tool, with higher ratings indicating higher perceived ease of use and user control domains. Results showed relatively high scores by participants with concussion, with the perceived ease of use being 4.42 (SD 0.46) and user control being 3.93 (SD 0.45) on a 5-point scale. Most participants were using the MyGuide tool on the desktop platform.

### Acceptance: Adherence

Both quantitative and qualitative data were used to understand adherence to using the tool. Survey data suggested that most participants had 1 to 5 visits to the tool in total. Furthermore, the survey data indicated that almost half of users completed 5 or more of the 11 modules.

### Impact

Based on the Health-ITUES measure, where a rating of 5 on a Likert scale indicates strongly agree, Perceived Usefulness was 4.10 (SD 0.57), Impact was 4.41 (SD 0.46), and Overall Usability was 4.27 (SD 0.35). These high scores indicate that the tool had a positive impact on self-management and quality of life. Participants with concussion reported that they were looking for strategies to manage their concussion symptoms (8/8, 100%), more information about concussion symptoms (7/8, 88%), specific strategies for returning to normal day-to-day activities (7/8, 88%), and information on how to navigate the health care system (5/8, 63%). Overall, survey data suggested that people with concussions were either satisfied (4/8, 50%) or very satisfied (3/8, 38%) with the tool and generally described that the tool had met their needs (6/8, 75%). Respondents indicated that the tool provided background information about concussion, provided ways to manage concussion symptoms, and motivated recovery. All survey respondents indicated they would recommend the tool to other people with concussions, as they noted it was informative and provided people with reassurance and normalization of symptoms being experienced.

### Qualitative Results

A total of 8 participants with concussions and 8 clinicians were included in the study. Participants with concussion included 4 females and 4 males with an average age of 33.8 years (SD 12.3; Table 1). Clinician participants included 7 occupational therapists and 1 physiotherapist, averaging 13.1 (SD 10.8) years in clinical practice with 8.5 (SD 4.7) years in concussion rehabilitation specifically (more details in Table 3).

**Table 3:** Demographics of clinician participants.

Characteristics		Participants, n (%)
**Profession**		
	Occupational therapist	7 (87.5)
	Physiotherapist	1 (12.5)
**Sector**		
	Private	7 (87.5)
	Public	1 (12.5)
Average years in practice, mean (SD)		13.1 (10.8)
Average years in concussion rehabilitation, mean (SD)		8.5 (4.7)
**Location**		
	BC Greater Vancouver	8 (100)

### Perceptions

#### Usability

Interview data provided context to the quantitative data. The majority of participants with concussions reported that the tool was simple and straightforward to use. One participant said, “[the website is] very linear... It shows you what you’ve done and, what you haven’t, and what you need to do next… It’s very, very well structured.” Participants also indicated that the clearly labeled topics, easy-to-understand section titles, and simple layout of the tool made it easy to find information. However, some participants reported confusion around use and navigation when initially using the tool independently, without the facilitation of clinicians.

Interview data from clinician participants indicated that the tool provided useful, curated, easy-to-understand, and credible information. One clinician explained:

Having one sort of a one-stop shop of information that's easy to go through is useful… [people with concussions] get lots of different information from different providers, and if they're symptomatic at the time, it's hard to take it all in…having somewhere just one source to go to is helpful. [The tool is] in an easy-to-read format, so it's not a lot of medical speak.

Some clinician participants mentioned that some sections had a lot of information, which may have made clients feel overwhelmed.

#### Accessibility

Participants seemed to show a preference for different visuals than others (eg, dark mode vs light mode, and font sizes), and many described the value of the individualization and customization of the tool. Participants also reported that the concussion-specific accessibility features inherent in the tool (eg, clear, short sections of information) were helpful in creating an accessible tool. One participant stated, “[The site] was very accessible…the sections were clear, they were short, you know, the content was good. You know, pretty hard to misunderstand.” Furthermore, participants noted specific features that might enhance accessibility (eg, audio presentation of information and optimization of the tool on a mobile platform).

#### Suitability

Participants with a concussion indicated that the tool was suited for people who had had a recent concussion or were in the early stages of recovery and seeking basic information about their concussion. The suggested time for access to the tool was within the first 6 weeks of injury to receive the maximum benefit as knowledge and information were most needed at that point. As one participant described,

I think it would be used more as, like a stepping stone... I think it's something I'd go to for just the very basics, or I didn't know what was going on with my health or understand my situation, my condition.

On the other hand, some clinician participants indicated that the MyGuide tool was appropriate for individuals with concussion at any stage of recovery. As one participant described their experience using the tool with different clients,

I’ve worked with people that are a week post-concussion to two years post-concussion, and I feel like it can work for everyone again, […] it’s just kind of a foundation. I think I am comfortable referring every single one of my concussion clients.

Clinician participants also noted that the tool could be suitable for individuals who were avoiding activities due to their concussion symptoms. These participants indicated that providing early education on topics like pacing was particularly important for these individuals, as the concept of pacing could be applied to various aspects of the person’s recovery process or therapies (eg, physiotherapy). One clinician explained:

I would refer patients [to the site] who are probably struggling with pacing…I feel like getting their pacing under control is usually a higher priority than giving them, you know, some form of manual therapy, that's my perspective…because it's that art of figuring out what to address at what point in time, often when they are so significantly concussed.

Clinician participants also indicated the different levels of suitability for some of their clients with chronic symptoms. As one clinician participant described:

Someone who’s really chronic, let’s say, post two years, oftentimes has built a pretty strong identity around their health... So I’m not sure that the impact would be the same per se, as someone earlier on in the experience. I think I’d still use it. It’s really hard to know what somebody has been exposed to in terms of education and material… And maybe they haven’t had any of that, or they have really low health literacy. So no, I still think it would be useful [but] in my experience, a lot of people have really built a disability identity by that point. And the concussion is something that has become part of who they are... if there's a tool like this might not be as impactful.

Clinician participants indicated that the tool may not be appropriate for clients who are feeling overwhelmed and anxious, having difficulty processing information, and experiencing no screen tolerance, low computer literacy, and poor English comprehension.

### Acceptance: Adherence

Based on the interview data, the primary reason people with concussions continued to actively engage in the tool was to seek out information to gain a better understanding of concussion recovery. Participants reported that they stopped using the tool when they acquired enough information or when they saw improvements in their recovery. One participant said of the tool, “It is sort of a learning tool, right?... But once I’ve learned how I can just focus on that myself, and I didn’t really return to it, but I was using what it had taught me.” Overall, the tool was often used over a short period of time and not used as a resource that participants with concussions were continuously referred to over a prolonged period.

Clinician participant perspectives gave further insight into other factors related to adherence. As most clinician participants noted they were actively referring their clients to the site, one factor that may have influenced their clients’ adherence was based on how they were directing clients to the site. Participants mentioned they were either referring clients to the tool by working through the tool with their clients in sessions or by sending the link to their clients to have them go through the tool independently at home. For some participants, going through the tool was a good starting point for their clients, particularly when doing virtual sessions. One clinician said, “It’s been really helpful for the clients when we do Zoom sessions... I can share my screen, and we can go through it together.”

Clinician participants mentioned that most clients with concussions were only engaging with the MyGuide Concussion tool a few times independently but mostly accessed the tool when facilitated by the clinician. One participant stated,

I get the sense that, similar to most things I give them, [the client] needs to be directed and guided... I would say if I just gave the person that website, I would not anticipate that they're just going through it all themselves. If there are specific parts of it that I would want them to get out of it, I would definitely follow up and make sure that they really got what I wanted them to get out of it.

As such, participants noted that the MyGuide Concussion tool was useful in therapy sessions but may not be as relevant if self-directed by participants.

### Impact

#### Overview

During the interviews, participants reported value in the tool’s information about various stages of concussion recovery. Participants with concussion noted that the tool was a trustworthy source of information, describing that affiliation with a predominant health care institute was helpful to increase the credibility of the tool. In addition, participants with concussions expressed that referral from clinicians or other people with concussions increased their acceptance of this tool as a credible source of information. Many clinician participants indicated that the tool reinforced the basic information about concussion recovery and concussion self-management that they were teaching their clients and helped the clients connect concepts important for their recovery.

Several participants with concussions reported that the self-assessment strategies in the tool helped them assess and become more aware of their triggers and were beneficial in helping alleviate symptoms. One participant expressed that the tool reminded them how everything is connected, referring to how their physical and psychological symptoms impact their ability to meet their life demands. The tool helped them manage their symptoms of low mood and find ways to meet their life demands while also balancing their other physical and psychological concussion symptoms. Clinician participants shared that the application of the tool decreased the risk of complications and potentially reduced the overall length of recovery. One participant said:

I generally see people that are at least six weeks, but generally more like three months post-concussion. And I would say the majority of them have gotten to that point because they’ve been avoiding all activity. And I think the tool does a really good job of letting people know that’s not what to do to get back and gives people a bit of a guide. So, I think that's kind of the reason that it could decrease the complications that we know happen once somebody is avoiding things for too long.

Many participants with concussions explained that while physical symptoms may be more clearly linked to concussion, the psychological impacts were not as recognized as symptoms of a concussion. As one participant described,

It help[ed] me to realize that a lot of the symptoms I’m experiencing weren’t from some other thing. It was from my concussion, even though it had happened quite a while ago. … so just being able to appreciate that these things are normal, and that... there's not something else wrong.

Another participant described their frustration of not knowing the cause of their symptoms to the point of questioning the validity of their symptoms. They reported that the information on the tool not only normalized their symptoms but also provided them with validation that their symptoms were indeed related to their concussion. Clinician participants similarly expressed that the tool helped their clients normalize the symptoms they were experiencing and provided reassurance to their clients.

Participants with concussion indicated various changes they made to managing their concussion symptoms or overall health based on what they learned from the tool, some of which included gradually returning to physical activity and improving their ability to manage stress. In contrast, other participants indicated that they had not made any changes to how they managed their symptoms because they had already made the necessary changes before finding the tool.

In the cases where clients were receiving services from multiple providers, some clinician participants noted that the MyGuide tool validated and reinforced the information provided to clients by different clinicians. For some other participants, the tool helped prime their client on a particular topic, which helped enhance the subsequent treatment session. In particular, one participant noted that not only did the tool provide opportunities to learn about concussion recovery outside of treatment time and enhance the client’s understanding of their condition, but it also allowed for the client to elicit social support from friends and family. Therefore, the tool both empowered clients to self-manage their concussions and encouraged reengagement with their social network and daily life activities.

Table 4 summarizes participants with concussion and clinician’s perspectives on the tool’s impact. Overall, data collected from the clinician participant interviews supported the perspectives of participants recovering from a concussion.

**Table 4:** Summary of perspectives on the impact of MyGuide on concussion recovery.

Participants with concussion	Clinician participants
Improved understanding of concussion symptoms and recovery process (from acute symptoms to returning to life after concussion).	Reinforced basic information about concussion recovery and self-management.
Improved awareness of and ability to self-manage triggers.	Decreased potential complications associated with concussion.
Provided reassurance and normalized symptoms.	Provided reassurance and normalized symptoms.
Changed overall approach to recovery.	Increased client empowerment.
Improved stress-management.	Provided opportunity to elicit support from family or friends.
Facilitated gradual return to activity.	Supported occupational reengagement.

#### Relevance

From the interviews, participants with concussions indicated that the tool was useful for gaining basic knowledge about concussion recovery and self-management strategies. However, many reported that they found the information on the tool to be repetitive to other information they had already read; as one participant described, “it was a lot of reiterating stuff I kind of already knew... it wasn’t really telling me anything new.” Other participants expressed that the tool lacked specific and detailed content on various topics, such as nutrition and differentiating an acute concussion from persistent postconcussion symptoms. Another participant suggested that the tool would have been more useful if it included a self-monitoring tool, where individuals could track their symptoms and strategies used to manage their symptoms, which could be shared with their health care team.

Some participants with concussions expressed how they had only found out about the tool once they were further along in their concussion recovery and indicated that had they known about the tool earlier, it would have been much more useful to them then. One person highlighted this when describing their experiences after their injury, saying, “It took me months just to figure out what it was I was dealing with. So, if I had gotten [the tool] right away, it would have been extremely useful for me.”

Another participant described why it would have been beneficial for them to have known about the tool earlier,

If I had had [the tool] immediately after having the concussion or shortly after [with]in the month... there would have been a lot less stress from having to deal with it, and not knowing what the cause of some symptoms were, and just being able to have a more well-developed understanding of what a concussion looks like.

Participants with a concussion also indicated that the information on the tool may be outdated and perceived the tool to be less relevant as a result. Participants mentioned the importance of updating the tool regularly as new concussion research is published.

Clinician participants indicated that the tool supported and enhanced the services they provided to their clients. In particular, participants noted that the tool provided preliminary and important information for recovery in a timely manner, a mixture of information for different learning styles and needs, a reinforcement of information learned during in-person sessions, and an opportunity for remedial learning outside of treatment time. Learning outside of clinic time provided opportunities for clients to lead and participate more in their own recovery. One clinician said, “I tend not to refer, I tend to work through the concussion site with clients, I tend not to just […] sort of give it to them.”

For some participants, it allowed them to focus on addressing more specific problems impacting recovery (eg, teaching the client about pacing). As one participant shared:

If it’s a motivated person, and they are actually reading through [the tool]... I don’t have to spend as much time necessarily providing some of that intro-level education. So, I think it can mean that we can move through treatment quicker. I like to give everyone the broad education, and then really more specific as we go to that person’s individual barriers. But if a lot of the intro general stuff can be covered on that website, and the person's actually going through it… then I can get more specific quicker, which… for a clinician, that’s great... it just means that I can do things faster.

With the increase in demand for e-health services, many clinician participants expressed that the tool was compatible with this delivery method. In specific cases, the MyGuide tool could accommodate and provide access to important education on concussion recovery for individuals with limited or no insurance coverage. Other benefits of using the tool included improving overall workflow, providing consistent information across other clinicians or existing concussion rehabilitation programs, and enhancing other therapies (eg, physiotherapy).

### Integration of Mixed Methods Results

Qualitative and quantitative results were integrated at the level of analysis. However, the accessibility, suitability, and relevance of the tool were only assessed using qualitative data. Multimedia Appendix 3 displays the integration of results using joint display.

## Discussion

### Principal Findings

Our study indicated that the MyGuide concussion self-management tool has good acceptance and perception by all participants (people with concussions and clinicians) using the tool.

Specific recommendations were provided by both participants with concussions and clinician participants to improve the ease of use of the MyGuide tool. Many clinicians requested that a downloadable PDF form be made for each module, as it would be beneficial to increase the accessibility of the tool for those with reduced screen tolerance. This format could also address the minor navigation issues that a handful of participants with concussions experienced when using the tool.

Participants also provided suggestions for increasing personalization and adherence to the tool. Participants with concussions reported that the use of the tool stopped when they acquired enough information or when they saw improvements in their recovery. Based on this, it would be beneficial to add more detail to modules, especially around nutrition, which one participant noted to be sparser. Clinician participants found that certain topics, such as return to sports, were irrelevant to the client’s recovery, and some information was inaccessible to those with low or no screen tolerance or low computer proficiency. To address this, the tool should be recommended to clinicians as something that should be adopted into practice so they can support clients in refining the tool to be more relevant to their own recovery goals. Furthermore, adding audio or visual modes of delivery as opposed to text could increase accessibility for individuals with lower screen tolerance.

### Comparison With Previous Work

Findings from our study indicated the benefits of the MyGuide Concussion tool; specifically, participants emphasized the importance of reassurance to people with concussions by normalizing their experiences. Previous research looking at brain injury found that reassurance was ranked as highly important when considering aspects of an intervention [35] and that normalization of emotions and behavior were important facilitators of self-acceptance and willingness to reach out for help when needed [36]. Furthermore, the MyGuide Concussion tool was seen as beneficial for identifying symptoms, which, in turn, helped with finding ways to manage triggers. A study exploring barriers and facilitators of reintegration after concussion found that lack of concussion education was a major barrier to returning to learning, suggesting that the information provided in the MyGuide tool may be a facilitator in returning to everyday life following concussion [37]. Furthermore, education on symptoms following a brain injury has been tied to higher self-awareness and positive experiences during interventions [36], as well as better outcomes [35].

Findings in our study indicated satisfaction and benefit with using a web-based tool for self-management of symptoms and overall recovery after concussion. Similar web-based interventions designed for facilitating recovery in adolescents with mild TBI have also found that web-based interventions were effective in improving executive functioning and functional disability, although this was identified by parents instead of the adolescents themselves [38]. Other studies on mTBI have identified the active coping style involved in self-management as being linked to improved recovery after concussion and improved pacing of return to activity [39]. Participants with concussion and clinicians in this study both agreed that the MyGuide tool was best for individuals in the early stages of recovery who require basic information about concussion and self-management. In particular, participants with concussions suggested that providing the tool within one month of injury would be most beneficial as this is when concussion education is essential to reduce stress related to understanding concussions and what they look like. In line with our findings, other web-based interventions for mild TBI have identified time as a key factor affecting the effectiveness of the intervention, as individuals do not have the same need for more information later in recovery [35,38,39]. One study described that receiving information during an optimal time of exposure was more crucial to maximizing symptom improvement as opposed to the frequency of use of the tool [38]. Several other studies have highlighted providing timely services after a concussion as being a key factor in the effectiveness of self-management websites [13,19].

The ease of use of the MyGuide tool was reported to be relatively high for participants, and aspects such as the linearity of the modules, simple layout, and easy-to-understand information were described as effective by participants. The use of desktop computers seemed to be the preferred medium for the use of the tool, and those using a mobile device voiced the need for improvement of the mobile platform. Usability studies on web-based tools for people with TBI have similarly found that simple interfaces are favored [40]. However, in contrast to our findings, several studies on digital health have found that participants prefer using mobile devices over desktop computers as more people have mobile devices, and most people keep their mobile phones with them at all times compared with their computers [40,41]. This contributes to quick access to the digital tool whenever they need it, rather than just at home. Although participants in the present study all resided in urban areas, the use of digital platforms for health-related self-management programs has been noted as beneficial for use by people remote from treatment centers [42] and especially in rural areas [43], suggesting that the MyGuide tool has the potential to have more far-reaching benefits than was discussed in this study.

### Limitations

There are 3 main limitations to this evaluation. First, this study had a small sample size, which may limit the ability to generalize the findings to the broader clinical population of those with concussion. However, data saturation was met during qualitative interviews, and the findings show preliminary support for the use of the MyGuide tool. Second, there may have been selection bias with participants with concussions as they required the ability to participate in an online survey and, as such, a higher capacity to engage with screens than other people with concussions. To mitigate this effect, participants had the option of completing the survey by telephone, but no participants requested a telephone survey option despite 75% (6/8) of participants indicating decreased screen tolerance in the questionnaires. This suggests further research may be required to understand whether decreased screen tolerance impacted module completion and to assess the interpretability of the results to the questionnaires that reported having enough information as a reason to stop completing the modules. Finally, occupational therapists were overrepresented as clinician participants, with no physicians involved in the study and the findings not necessarily representing all clinicians. However, these findings provide crucial information for later studies that aim to specifically understand other clinicians’ perspectives.

### Future Research

Future research should be done to assess whether the tool can be used in practice by clinicians other than occupational therapists and if the earlier introduction of the tool can provide increased benefits to people with TBI. The use of the MyGuide Concussion tool should also be examined in the senior population, considering the frequency of concussions due to falls in this age category and because this study may have limited generalizability as it focused exclusively on young and middle-aged adults. In addition, it would be beneficial to gauge to what degree screen tolerance impacts the completion of modules and the need for clinician involvement. Finally, as this study revealed a lower rating for User Control relative to other features, it would be beneficial to explore what contributes to this lower rating and what improvements can be made to mitigate any difficulties.

The MyGuide tool is a web-based customizable concussion self-management tool with good acceptability and perceptions by people with concussions and clinicians in concussion rehabilitation. MyGuide provides foundational information on concussion and self-management strategies, normalizes symptoms, and provides reassurance and support for occupational reengagement.

## Appendix

Multimedia Appendix 1 Interview questions for client users.

Multimedia Appendix 2 Health care provider interview questions.

Multimedia Appendix 3 Integrated results matrix.

## Abbreviations

DEDHI: Design and Evaluation of Digital Health Interventions
Health-ITUES: Health Information Technology Usability Evaluation Scale
TBI: traumatic brain injury
