# Supplementary material for: The MyGuide Web-Based Self-Management Tool for Concussion Rehabilitation: Mixed Methods Cross-Sectional Study
Source: JMIR Rehabil Assist Technol. 2025 Jan 7;12:e59181. doi: 10.2196/59181 (PMC11751642; doi:10.2196/59181)
Supplement: Multimedia Appendix 1 [file rehab_v12i1e59181_app1.docx]

1. How did you hear about the MyGuide website?
2. Have you been using the website regularly?
   1. If you have, how often have you been using and how long are each of the sessions?
   2. If you have not, what has affected your ability to use the website regularly?
3. To what extent was the MyGuide website useful for you in your recovery, if it was at all?
   1. PROBE: How useful did you find this website was for your purposes?
4. How relevant was this website to you personally?
   1. PROBE: To what extent did the MyGuide website address the information needs or questions that you had related to managing your recovery?
   2. PROBE: What made it relevant or not relevant to you? How useful would this website have been to you sooner after the injury?
5. How did using this website affect your understanding about your concussion and your experiences with concussion symptoms, if at all?
6. What changes to the management of your concussion symptoms or care of your overall health, if any, did you make because of what you learned from this website?
   1. PROBE: Did you do anything differently in managing your concussion symptoms or caring for your overall health because of what you learned from the website?
7. How easy or difficult was it for you to understand information on this website?
8. How easy or difficult was this website to use?
   1. PROBE: Can you identify any challenges that you had using this website?
9. What was your experience like finding information on this website?
   1. PROBE: For instance, if you were looking for something specific, what was your experience like finding that information?
10. Do you have any comments related to accessibility? OR How accessible did you find this website to be?
    1. PROBE: Were you aware of any specialized features that were unique to the website like the ability to adjust the screen brightness and adjust the font size? If so, which features did you use when using the website and how did that impact your experience?
11. Are there any other accessibility features that were missing that would improve your overall experience when using the website?
12. How credible did you find this website to be?
    1. PROBE: What about this website made you feel it was credible or not credible?
13. What would you consider the strengths of this website to be?
14. What would you consider the weaknesses of this website to be?
15. Overall, how satisfied were you with this website?
    1. PROBE: What about this website made you satisfied or unsatisfied?
16. Have you been able to access any other concussion rehabilitation services where you live?
17. Do you have any suggestions for improving the website? Any other comments?
