# Supplementary material for: The MyGuide Web-Based Self-Management Tool for Concussion Rehabilitation: Mixed Methods Cross-Sectional Study
Source: JMIR Rehabil Assist Technol. 2025 Jan 7;12:e59181. doi: 10.2196/59181 (PMC11751642; doi:10.2196/59181)
Supplement: Multimedia Appendix 2 [file rehab_v12i1e59181_app2.docx]

1. What is your profession? What role do you have in concussion rehabilitation?
2. How many years have you been in practice in total?
3. How long have you been working in concussion rehabilitation?
4. What city is your practice located in?
5. Are you currently working for a health authority or private practice? a. If you are working for a health authority, which health authority are you employed with?
6. How did you hear about the MyGuide website?
7. Do you refer clients to the MyGuide website?
   1. PROBE: If you have been or haven’t been referring clients to the website, why?

***[If healthcare provider has been referring clients to the site or has been referring clients to the site at some point, continue to question #8]***

***[If the healthcare provider has not been referring any clients to the site, proceed to question #11]***

1. How have you been referring your clients to the site?
2. How has the website been received by your clients?
   1. PROBE: What are client’s attitudes towards the site?
   2. PROBE: Have the clients been using the website regularly?
3. What impact, if any, do you believe the My Guide website had on your clients’ recovery after concussion?
   1. PROBE: Could you describe any changes to the client’s self-management, symptom management or functioning as a result of using the website?
   2. PROBE: Could you describe any changes to the client’s physical/social/psychological/emotional outcomes as a result of using the website?
   3. PROBE: Could you describe any changes to the client’s attitudes or motivation about their recovery as a result of using the website?
   4. PROBE: Did you notice any impact on your clients’ ability or confidence to take action with lifestyle or behavior changes beneficial for their recovery as a result of using the website?
   5. PROBE: Could you describe any changes to the overall length of the recovery (as a result of using the website)?
4. Could you please describe the type of clients you would refer to the site and when it would be appropriate for you to refer the client to the site?
   1. PROBE: In what cases would you not refer clients to the website?
   2. PROBE: Under what circumstances/situations do you refer clients to the website?
   3. PROBE: What type of client is the website best suited for? What type of client is the website not suited for?
5. In general, what are your own impressions or thoughts about the MyGuide website?
   1. PROBE: Are there any particular features or resources on the site that you find useful/helpful as a healthcare provider? Are there any features or resources that you do not find valuable/helpful as a healthcare provider?
6. Overall, how might the website fit or not fit with your practice?
7. As a healthcare provider, what are the advantages or disadvantages of using the MyGuide website with your clients?
8. Has the MyGuide website changed your capacity or ability to provide services to support your client’s recovery in any way?
   1. If so, how has the website changed your capacity or ability to provide services to support your clients?
      1. PROBE: Are there any changes to the quality of the services provided? Please describe.
   2. If not, what changes to the website could be made to improve your capacity or ability to provide services to support your clients better?
9. Do you have any suggestions for improving the website so that it could be integrated more into clinical practice?
10. Do you have any recommendations on promoting the MyGuide website or “getting the word out” to other healthcare providers about using the website as a resource with their clients?
11. Do you have any other suggestions on how the site could be improved to better support your practice or the services you provide to your clients?
12. Are there any comments or suggestions you would like to share?
