# Supplementary material for: The MyGuide Web-Based Self-Management Tool for Concussion Rehabilitation: Mixed Methods Cross-Sectional Study
Source: JMIR Rehabil Assist Technol. 2025 Jan 7;12:e59181. doi: 10.2196/59181 (PMC11751642; doi:10.2196/59181)
Supplement: Multimedia Appendix 3 [file rehab_v12i1e59181_app3.docx]

| **Quantitative Results** | **Qualitative Results** | **Exemplar quote** |
| --- | --- | --- |
|  |  |  |
| Perceived ease of use ratings were relatively high | - Topics and section titles were clearly labelled and easy to understand, which increased accessibility for people with concussion - Layout was simple, making the tool straightforward to use - Some confusion with use and navigation initially | Participant with concussion: “[the website is] very linear... It shows you what you've done and what you haven't, and what you need to do next… It's very, very well structured.” |
| User control ratings were relatively high | - Information was useful, easy-to-understand, and credible - Amount of information in certain sections may be overwhelming for some clients of clinician participants | Clinician participant: “[people with concussion] get lots of different information from different providers, and if they're symptomatic at the time, it's hard to take it all in…having somewhere just one source to go to is is helpful” |
| Average total number of visits to the tool was 1-5 visits | - Use of tool was stopped when participants acquired enough information or when they saw improvements in recovery - Used more often during a short, critical period immediately after recovery when information was most needed, rather than as a tool to regularly refer back to | Participant with concussion: “It is sort of a learning tool, right?... But once I've learned how I can just focus on that myself, and I didn't really return to it, but I was using what it had taught me.” |
| Average number of completed modules was five or more out of eleven modules | - Clinician participants suggest adherence may have depended on clients’ learning style (independent vs. guided) and how tool is referred to clients (sending a link vs. facilitating use of the tool during sessions) - Generally clinician participants noticed better adherence when used during therapy sessions rather than when self-paced | Clinician participant: “I get the sense that, similar to most things I give them, [the client] needs to be directed and guided... I would say if I just gave the person that website, I would not anticipate that they're just going through it all themselves.” |
| Perceived usefulness, impact, and overall usability were rated relatively high | - Provides valuable information about various stages of concussion recovery and self-management - Affiliation of tool with a healthcare institute increased perceived trustworthiness and reliability of information - Referral by a clinician increased the tool’s perceived credibility - Beneficial in reinforcing information being taught during clinical sessions - Application of tool has potential to reduce complications and length of recovery | Clinician participant: “…I think the tool does a really good job of letting people know that’s not what to do to get back and gives people a bit of a guide. So, I think that's kind of the reason that it could decrease the complications that we know happen once somebody is avoiding things for too long.” |
| The tool was mainly accessed by participants for information in three areas: symptom-management, general concussion symptom information, and guidance on returning to normal day-to-day activities | - Improved awareness of symptom triggers, empowered self-management, and guided stress-management - Provided opportunity to elicit support from family/friends - Increased understanding of recovery process and interconnectedness of physical and psychological symptoms - Provided reassurance and normalization of symptoms - Facilitated return to activity (work, sports, etc.) - Reinforced information provided by clinicians regarding recovery and self-management | Participant with concussion: “It help[ed] me to realize that a lot of the symptoms I'm experiencing weren't from some other thing. It was from my concussion, even though it had happened quite a while ago. … so just being able to appreciate that these things are normal, and that... there's not something else wrong.” |
